# Driven by engagement and social identity: refining the SOR framework to explore digital music consumption behaviors among Chinese undergraduate students on short video platforms

**DOI:** 10.3389/fpsyg.2026.1758762

**Published:** 2026-03-06

**Authors:** Yu Shan, Yabo Xie, Zhan Li, Pujing Feng

**Affiliations:** 1 School of Music and Dance, Xihua University, Chengdu, China; 2 School of Education, Mianyang Teachers' College, Mianyang, China

**Keywords:** college students, digital music consumption, music recommendation satisfaction, short video platforms, social identity, Stimulus-Organism-Response (SOR) framework

## Abstract

Grounded in the Stimulus-Organism-Response (SOR) framework, this study refines the model within the context of short video platforms (SVPs) and digital music consumption. By incorporating social identity as a context-specific “Organism” mediator alongside music recommendation satisfaction, we explore the dual psychological pathways-individual and social-through which SVP usage shapes the consumption behaviors of Chinese undergraduates. We identify four key “Stimulus” variables (platform usability, social influence, emotional regulation, algorithm perception) and measure digital music consumption behavior as the “Response.” Data were collected from 602 Chinese undergraduates via a mixed-method survey and analyzed using SPSS 23.0 and AMOS 21.0. The results demonstrated strong scale reliability (all Cronbach's α > 0.8) and validity (KMO = 0.923; CFA indicated good fit: χ^2^/df = 1.415, GFI = 0.952, CFI = 0.988, RMSEA = 0.032). Correlation analysis revealed significant positive relationships (*r* = 0.4–0.7, *p* < 0.001) among key variables. Regression and bootstrap mediation analyses confirmed that: (1) the four stimulus factors are positively associated with consumption behavior; (2) music recommendation satisfaction mediates the effects of platform usability, emotional regulation, and algorithm perception (standardized indirect effects = 0.202, 0.207, 0.183, *p* < 0.05); (3) social identity specifically mediates the relationship between social influence and consumption (standardized indirect effect = 0.245, *p* < 0.001); (4) the indirect effect of social influence through music recommendation satisfaction is not significant (standardized indirect effect = 0.005, 95% CI [-0.031, 0.033]); (5) social influence emerged as the strongest predictor (β = 0.27 in SEM, β = 0.182 in regression, *p* < 0.001). The refined model explained 58.2% of the variance in consumption behavior and 32.9% in recommendation satisfaction. These findings deepen the understanding of context-bound psychological mechanisms in SVP-driven music consumption and offer practical insights for platform operators, industry stakeholders, and educators.

## 1 Introduction

Short video platforms (SVPs) have become ubiquitous social media tools in the mobile internet era, characterized by low entry barriers, high penetration rates, and strong interactivity—features that make them powerful disseminators of digital music (Bell et al., 2023; Bhargava, 2023). Driven by the widespread adoption of smartphones and advances in internet technology, the number of global internet users has grown exponentially (Ou et al., 2022), and college students—who are the core consumer group of digital music—are increasingly relying on SVPs to access, discover, and share music (Radesky et al., 2022). Unlike traditional music platforms, SVPs integrate entertainment, social interaction, and music dissemination through a “text + image + audio-video” format. This integration not only meets college students' needs to explore new music and express their musical preferences but also fosters a sense of social belonging by allowing them to participate in music challenges, share BGM (background music) with peers, and construct musical identities (Maccarrone-Eaglen and Schofield, 2023).

Thus, investigating how SVPs shape college students‘ digital music consumption behavior is both theoretically and practically meaningful. Theoretically, this study extends the application of the SOR framework to the context of SVP-based music consumption by introducing social identity as a key mediator, addressing the gap that existing SOR studies have overlooked the social nature of young consumers' music behavior (Park and Jung, 2024). It also enriches the literature on digital music consumption by clarifying the dual psychological pathways (individual satisfaction and social identity) driving consumption. Practically, the findings provide actionable insights for three stakeholders: (1) SVP operators: optimize platform functions to enhance user experience and social interaction; (2) digital music industry practitioners: develop targeted marketing strategies to convert SVP exposure into consumption; (3) university educators: design media literacy programs to guide students toward rational music consumption. Given these contributions, this study addresses an important and understudied topic in current media and consumer psychology research.

Recent studies on SVPs and digital music consumption have adopted various methodologies, such as tracking user-platform interactions (Lowenstein-Barkai and Lev-On, 2022) or mining large-scale behavioral data via artificial intelligence (Alasa et al., 2025). While these studies effectively identify behavioral correlations, they fail to unpack the psychological mechanisms that link SVP use to music consumption decisions. The SOR framework has been widely used to explain user behavior on SVPs, such as binge-scrolling (Nunes and Birdsall, 2022), but existing applications have two key limitations: (1) the “Organism” component is often simplified to individual cognitive or emotional states (e.g., satisfaction) without considering the social nature of music consumption among young adults; (2) the set of “Stimulus” variables is often narrow, lacking a comprehensive integration of platform, social, and emotional factors. Although some scholars have proposed models including content, source, and platform credibility (Katyal and Sehgal, 2025), these frameworks lack robust empirical validation in the context of digital music. This leaves a critical research gap: there is no empirical study that integrates comprehensive stimuli into an extended SOR framework to explain the dual psychological pathways (individual and social) of college students' digital music consumption on SVPs. To fill this gap, this study extends the SOR framework by adding social identity as a second “Organism” mediator. We define the “Stimulus” (S) as four factors (platform usability, social influence, emotional regulation, algorithm perception), the “Organism” (O) as two parallel mediators (music recommendation satisfaction, social identity), and the “Response” (R) as digital music consumption behavior. Using a questionnaire survey of 602 college students and multiple statistical methods (correlation analysis, regression analysis, bootstrap mediation analysis, confirmatory factor analysis), this study aims to: (1) test the direct effects of the four stimulus factors on consumption behavior; (2) verify the mediating roles of music recommendation satisfaction and social identity; (3) clarify the most influential factors driving consumption behavior. This design ensures theoretical depth and methodological rigor, leading to more generalizable findings.

To empirically test this comprehensive model, data were collected through a questionnaire survey administered to a large sample of university students. The proposed hypotheses were examined using regression analysis, which allows for the quantification of both the direct effects of the stimulus variables and the indirect effects mediated through recommendation satisfaction. This methodological approach is positioned to offer a more integrated understanding of the psychological mechanisms linking SVP engagement to music consumption. The findings are expected to contribute to the theoretical development of the SOR framework in digital media contexts and provide actionable insights for practitioners in the music and platform industries.

## 2 Research model and hypotheses

### 2.1 Research model of the impact of SVPs on university students' music consumption behavior

This study extends the SOR framework to analyze the influence of SVPs on college students' digital music consumption behavior. Originating from psychology and behavioral science, the SOR framework explains how external “Stimulus” (S) triggers internal psychological processes in the “Organism” (O), which in turn leads to “Response” (R) behaviors (Park and Jung, 2024). Based on the characteristics of SVP music consumption and social identity theory (Tajfel and Turner, 1979), we divide the influence process into three stages: Stimulus stage (S): External factors from SVPs that trigger college students' psychological responses, including four variables: platform usability (user-friendly interface and functions), social influence (external social cues and normative pressure, such as peer sharing, celebrity recommendations, music charts), emotional regulation (music's ability to match and adjust mood), and algorithm perception (accuracy and intelligence of music recommendations). Organism stage (O): Internal psychological processes mediating between stimulus and response, including two parallel mediators: (1) music recommendation satisfaction (subjective evaluation of recommended music's style match, quality, and timeliness); (2) social identity (internalized self-concept and sense of belonging derived from participating in SVP music activities, such as sharing BGM or joining music challenges, including identification with peer groups, self-categorization based on musical tastes, and integration of musical preferences into personal identity). Behavioral response stage (R): Specific digital music consumption behaviors of college students, including purchasing platform memberships, digital albums, singles, rewarding creators, and following musicians (Kok et al., 2025).

In the field of digital music marketing, SOR theory can be used to explain and predict university students' music consumption behavior responses on SVPs. Extensive research indicates that environmental stimuli from SVPs play a crucial role in university students' musical emotional experiences, musical cognitive construction, and actual consumption behaviors. For example, personalized digital music recommendation ads can significantly enhance university students' favorability toward music brands, thereby substantially increasing their purchase intentions. With the rapid development of social media and the continuous advancement of real-time data analysis tools, music brands and SVPs can more accurately discern university students' musical preferences, emotional resonance points, and cognitive patterns. Based on this in-depth understanding, brands and platforms can develop more targeted personalized marketing strategies, monitor university students‘ music interaction behavior data on the platform in real time (Abdul et al., 2023), including the viewing time of different music short videos, likes, and comments, and flexibly adjust music advertising content and placement strategies accordingly to effectively improve music promotion effectiveness. The application of SOR theory in short video digital music marketing not only helps marketers optimize music-related short video content to better align with university students' musical preferences and psychological needs, thereby improving the precision and influence of music marketing, but also enables companies to gain a deeper understanding of the motivations, emotions, and cognitive factors underlying university students‘ music consumption behavior, enabling more accurate predictions of their consumption trends (Melnyk et al., 2022), and laying a solid theoretical foundation for further research into the impact of SVPs on university students' digital music consumption behavior.

**Figure 1:**
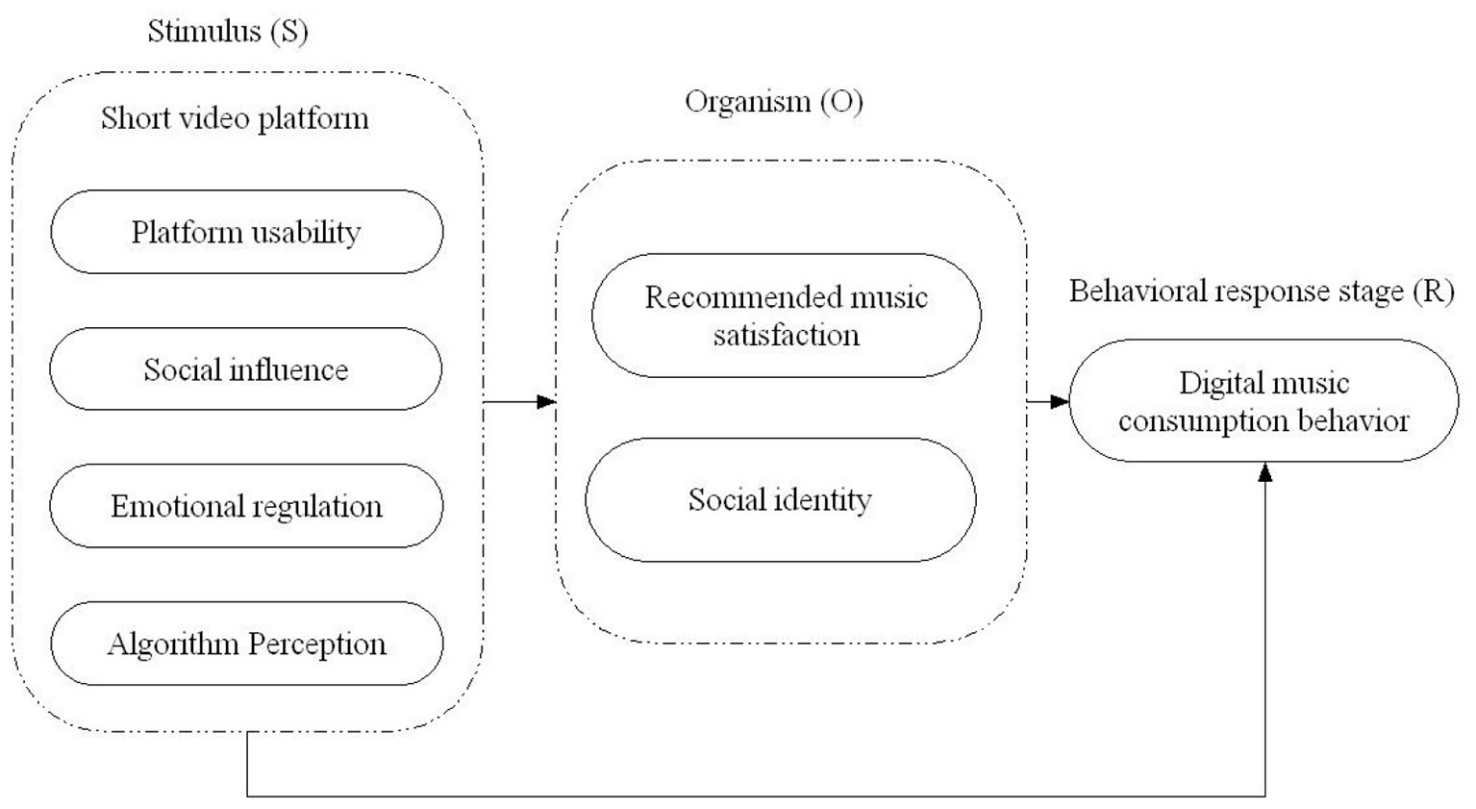
Research model on the influence of SVPs on university students' digital music consumption behavior.

Therefore, based on the analysis of the influence of SVPs on university students' digital music consumption behavior using the SOR model, a research model is constructed, as shown in Figure 1. Among them, platform usability, social influence, emotional regulation, and algorithm perception are independent variables, representing different aspects of SVPs that stimulate university students. Music recommendation satisfaction is the mediating variable, reflecting university students' subjective evaluation and feelings toward the music recommended by the platform. Digital music consumption behavior serves as the dependent variable, reflecting university students' final consumption decisions and actions in the digital music domain.

Conceptually, social influence and social identity are distinct: social influence is an external stimulus (e.g., peer sharing, celebrity recommendations) that triggers psychological responses, while social identity is an internal psychological state (e.g., belongingness, self-categorization) formed by processing these external stimuli. Although social identity items involve ‘peers' or ‘social circles', they measure the internal psychological outcomes of social influence, rather than the external stimuli themselves. Statistical discriminant validity (square root of AVE > inter-construct correlations, Table B1) confirms their distinctiveness. We modeled music recommendation satisfaction and social identity as parallel mediators based on theoretical and practical considerations. Theoretically, music recommendation satisfaction reflects individual cognitive evaluations of the platform's music services, while social identity reflects collective psychological states formed through social interactions. These two psychological mechanisms operate independently: individual satisfaction is driven by personal platform experiences, while social identity is triggered by social influence. Practically, prior studies on digital music consumption have shown that individual preferences and social factors are distinct drivers with no clear sequential relationship, supporting the choice of parallel mediation.

The variables in the model are described as follows:

(1) Independent variables

Platform usability: This encompasses the user-friendliness of the interface design, such as whether operations are simple, function buttons are easy to identify and click (Inoue et al., 2022); the clarity of navigation, such as whether music categories can be found quickly and search results are accurately displayed; and the practicality of music functions, such as whether music playback controls are convenient and whether functions such as adding to favorites and creating playlists are easy to use. These aspects collectively constitute platform usability, which influences university students' experience of accessing music on the platform.

Social influence: This refers to external social cues and normative pressure from SVPs, including the music sharing behavior of friends on social media platforms (e.g., recommending favorite music through posts or private messages), the guiding role of popular music charts (e.g., university students may pay attention to and try new music because of chart rankings), and the influence of music recommendations by popular bloggers (e.g., their promotion and sharing of listening experiences may change university students' perceptions and interests in music) (Kim et al., 2022).

Emotional regulation: This involves the compatibility of music with university students' different emotional states. When university students are anxious or tense, soothing classical music or light instrumental music may have a relaxing effect; when they are excited or happy, upbeat pop music can enhance their sense of pleasure. This regulatory function of music on emotions influences university students' demand for and evaluation of music.

Algorithm perception: This mainly refers to the accuracy and intelligence of music recommendations made by short video platform algorithms based on university students' historical behavior data (such as playback records, collection preferences, search keywords, etc.). Whether algorithms can accurately capture changes in university students' musical tastes and recommend music that both aligns with their current interests and has a certain degree of novelty (Purnama et al., 2024) will influence university students' trust and recognition of the algorithms.

(2) Mediating variables

Music Recommendation Satisfaction: This is the comprehensive evaluation of university students' satisfaction with the music recommended by short video platforms across multiple dimensions. This includes the match of music styles, i.e., whether the recommended music covers university students' favorite genres such as pop, rock, and folk; music quality, such as whether the sound quality is clear and the arrangement is well-crafted (Liyanage and Balalle, 2024); and the timeliness of recommendations, i.e., whether appropriate music is pushed in a timely manner when university students have new music needs. This satisfaction reflects university students' internal acceptance and recognition of the platform's music recommendations.

Social Identity: Social identity refers to the internalized self-concept and sense of belonging that college students gain through participating in music-related activities on SVPs (Tajfel and Turner, 1979). It includes: (1) identifying with peer groups that share musical preferences; (2) gaining a sense of belonging through music challenges or BGM sharing; (3) regarding musical taste as part of one's digital identity on SVPs. This psychological state is triggered by social influence and further correlates with digital music consumption behavior.

(3) Dependent variable

Digital music consumption behavior: manifested in university students' actual consumption decisions and actions related to music. Specifically, this includes purchasing membership services on music platforms to access more music resources and privileges (Kok et al., 2025); paying for digital albums to support favorite artists or music works; purchasing singles to meet specific listening needs; and participating in music tipping activities to express support for high-quality music creators, among others.

### 2.2 Research hypotheses

#### 2.2.1 The impact of independent variables on mediating variables

Platform usability and satisfaction with recommended music: When the user interface of a short video platform is simple and clear, with a reasonable layout and easy-to-use music-related functions such as search and bookmarking, university students can more easily discover music that suits their preferences. This convenient user experience will increase their satisfaction with the music recommended by the platform. Therefore, we hypothesize that:

H1: Platform usability has a significant positive effect on satisfaction with recommended music.

Social influence and satisfaction with recommended music: On SVPs, social factors such as music shared by friends, rankings on popular music charts, and recommendations of specific music by influencers can influence university students' perceptions of music preferences. If their social circles give positive feedback on a certain type of recommended music, university students are more likely to accept these recommendations, thereby increasing their satisfaction with the recommended music. Therefore, we hypothesize that:

H2: Social influence has a significant positive effect on satisfaction with recommended music.

Emotional regulation and satisfaction with recommended music: university students use music on SVPs to regulate their emotions. If the music matches their current emotional state, it will resonate with them (Nunes and Birdsall, 2022). This emotional satisfaction will cause university students to give higher ratings to the music recommended by the platform, thereby increasing their satisfaction. Therefore, we hypothesize that:

H3: Emotional regulation has a significant positive effect on satisfaction with recommended music.

Algorithm perception and satisfaction with recommended music: If the algorithm of a short video platform can accurately identify university students' music preferences based on their past listening history, search records, etc., and continuously push music that matches their interests, and can adjust the recommended content in a timely manner according to the dynamic changes in their interests, university students will believe that the algorithm understands them, thereby increasing their satisfaction with the recommended music. Therefore, we hypothesize that:

H4: Algorithm perception has a significant positive effect on recommended music satisfaction.

#### 2.2.2 The influence of mediating variables on the dependent variable

When university students are highly satisfied with the music recommended by SVPs, it means that the recommended music aligns with their expectations in terms of style, quality, and other aspects. This enhances their trust and reliance on the platform's music recommendations. Based on this, they are more likely to engage in music consumption behaviors such as purchasing platform memberships, paying for digital albums, or buying individual tracks due to the platform's recommendations (Ning et al., 2022). Therefore, we hypothesize:

H5: Satisfaction with recommended music has a significant positive effect on music consumption behavior.

#### 2.2.3 The influence of independent variables on dependent variables

Platform usability and digital music consumption behavior: A user-friendly short video platform with convenient operations and functional features can reduce the time and effort university students spend accessing music. This positive user experience enhances their willingness to engage in music consumption on the platform, prompting them to purchase music products more frequently and participate in music-related paid activities. Therefore, we hypothesize:

H6: Platform usability has a significant positive impact on digital music consumption behavior.

Social influence and digital music consumption behavior: Music sharing, recommendations, and discussions within social circles may trigger university students' conformity or curiosity, leading them to engage in music consumption. Therefore, we hypothesize:

H7: Social influence has a significant positive impact on digital music consumption behavior.

Emotional Regulation and Music Consumption Behavior: Music's effective regulation of university students' emotions leads them to develop a deeper emotional dependence on music. To sustain this emotional fulfillment, they are more willing to pay for music. Therefore, we hypothesize that:

H8: Emotional regulation has a significant positive effect on digital music consumption behavior.

Algorithm Perception and Music Consumption Behavior: Precise algorithmic recommendations can continuously uncover and satisfy university students' latent music needs, enabling them to discover more music that aligns with their preferences. When university students perceive that algorithm understand their musical tastes, they are more likely to trust the platform's recommendations and, consequently, more willing to pay for recommended music. Therefore, we hypothesize:

H9: Algorithm perception has a significant positive influence on digital music consumption behavior.

#### 2.2.4 The impact of independent variables on the new mediating variable (social identity)

Social influence and social identity: On SVPs, social factors such as music sharing by friends, recommendations by internet celebrities, and music chart rankings can promote college students to integrate their musical preferences into group norms (Kim et al., 2022). Participating in music challenges or using popular BGM can help them gain recognition from peers, thereby enhancing their sense of social identity. Therefore, we hypothesize that:

H10: Social influence has a significant positive effect on social identity.

#### 2.2.5 The impact of the new mediating variable on the dependent variable

When college students form a strong social identity through SVP music activities, they are more likely to consume music to maintain their group identity (Maccarrone-Eaglen and Schofield, 2023). For example, purchasing digital albums recommended by peers or supporting musicians favored by their social circle to strengthen their sense of belonging. Therefore, we hypothesize that:

H11: Social identity has a significant positive effect on digital music consumption behavior.

#### 2.2.6 The mediating role of the new mediating variable

Social influence can indirectly affect digital music consumption behavior by enhancing college students' social identity (Tajfel and Turner, 1979). That is, social influence first promotes the formation of social identity, and then social identity correlates with consumption behavior. Therefore, we hypothesize that:

H12: Social identity mediates the relationship between social influence and digital music consumption behavior.

#### 2.2.7 The moderating role of social identity

Social identity may strengthen the positive association between music recommendation satisfaction and digital music consumption behavior. College students with higher social identity are more likely to translate their satisfaction with recommended music into consumption, as the music not only meets personal preferences but also reinforces group identity. Therefore, we hypothesize that:

H13: Social identity moderates the relationship between music recommendation satisfaction and digital music consumption behavior, such that the positive association between music recommendation satisfaction and consumption behavior is stronger for college students with higher social identity.

## 3 Survey questionnaire design and formal research

### 3.1 Questionnaire design

The purpose of designing the survey questionnaire is to collect research data to explore which factors influence university students' digital music consumption behavior on SVPs. The questionnaire content fully considers the characteristics of university students as digital music consumers and is divided into three parts:

(1) Questionnaire description: At the beginning of the questionnaire, the identity of the investigator is stated, the background, significance, and purpose of the survey are explained, it is noted that the survey is conducted anonymously, and the data collected is only for academic research.
(2) Basic information of respondents: In addition to collecting personal information such as gender, age, grade, and monthly family income, there is a specific investigation into the total duration of short video usage by university students.
(3) Analysis of influencing factors: This part serves as the key section of the questionnaire, investigating the impact of factors such as platform usability, social influence, emotional regulation, and algorithm perception on university students' digital music consumption behavior, as well as their satisfaction with recommended music and actual music consumption behavior. This section uses a five-point Likert scale, where 1 means “strongly disagree;” 2 means “disagree;” 3 means “neutral;” 4 means “agree;” and 5 means “strongly agree,” allowing university students to score based on their actual feelings.

Items were adapted from existing literature and revised for the SVP context: (1) Platform usability was adapted from Inoue et al. (2022); (2) Social influence was adapted from Kim et al. (2022); (3) Emotional regulation was adapted from Nunes and Birdsall (2022); (4) Algorithm perception was adapted from Purnama et al. (2024); (5) Music recommendation satisfaction was adapted from Liyanage and Balalle (2024); (6) Social identity was adapted from Tajfel and Turner (1979) and Maccarrone-Eaglen and Schofield (2023); (7) Digital music consumption behavior was adapted from Kok et al. (2025). Three experts in media psychology and consumer behavior reviewed the questionnaire for content validity, and wording of ambiguous items was revised. A pilot test with 80 college students confirmed acceptable reliability (Cronbach's α > 0.8) and construct validity (factor loadings > 0.6).

The design of the survey questionnaire is shown in Table 1.

**Table 1:** Survey questionnaire design and standardized factor loadings of each construct.

**Variable**	**Question number**	**Title**	**Standardized factor loading**
Platform usability	1	I can easily learn to use the music related functions of short video platforms	0.765
	2	The process of discovering new music through short videos is simple and intuitive	0.792
	3	The platform interface design allows me to quickly find music content	0.813
	4	The synchronization function between video and music (such as “shoot the same style”) is easy to operate	0.748
	5	The threshold for using music special effects/filters is relatively low	0.697
	6	Clear and precise classification of platform music (such as “Guofeng” and “Dianyin”)	0.784
	7	Easy steps from short video to music purchase/collection	0.821
	8	The platform's music search function (such as humming recognition) is accurate and efficient	0.773
Social influence	9	I will imitate the music my friends use in short videos	0.759
	10	The platform music charts (such as the “Hot Song Chart”) influence my choices	0.732
	11	Seeing multiple people using the same BGM increases my willingness to use it	0.705
	12	I will gain more social interaction (likes/comments) through music related short videos	0.768
	13	Participating in music challenges can enhance my sense of social belonging	0.689
	14	I will share videos with specific BGM to express my personal attitude	0.724
	15	Music preference has become a part of my social identity on short video platforms	0.716
	16	I will pay attention to the background music used by internet celebrities/stars	0.743
	17	The music like records of platform friends will affect my consumption	0.701
	18	Using popular BGM can increase my video exposure	0.786
Emotional regulation	19	I will choose different styles of music short videos based on my mood (such as listening to light music before bedtime)	0.835
	20	When under a lot of pressure, watching music videos can help me relax	0.807
	21	The music recommended by the platform's “emotion recognition” usually matches my current state	0.752
	22	I will collect specific BGMs for future emotional scenarios (such as during exercise)	0.739
	23	Watching short music videos is one of the ways I regulate my sense of loneliness	0.718
	24	Different short video performances of the same song can trigger different emotional reactions in me	0.764
	25	I will express my current state of mind by posting short videos with BGM	0.826
	26	The content of the “healing music” tag on the platform can effectively alleviate my anxiety	0.811
	27	Repeatedly watching short videos of my favorite music can enhance my positive emotions	0.794
	28	Short music videos can trigger my emotional resonance more than pure audio	0.803
Algorithm Perception	29	I noticed that short video platforms recommend content based on my browsing history.	0.842
	30	The short videos recommended to me usually align with my personal interests.	0.727
	31	I realized that platform algorithms will prioritize pushing popular or highly interactive content.	0.682
	32	I feel that the content recommended by algorithms is becoming increasingly‘ homogeneous' (repeating similar topics).	0.838
Recommended music satisfaction	33	I am generally satisfied with the music recommended to me by short video platforms.	0.854
	34	The music recommended by the platform is in line with my personal taste.	0.796
	35	The recommended music genres are sufficiently diverse (such as pop, rock, electronics, etc.).	0.819
	36	I often discover new favorite music or musicians through short videos.	0.775
	37	The frequency of recommended music updates (such as daily recommendations) keeps me interested.	0.808
	38	The platform can accurately understand changes in my music preferences (such as recent interest shifts).	0.761
Music consumption behavior	39	I will purchase or subscribe to music platform memberships (such as QQ Music, NetEase Cloud) due to short video recommendations.	0.733
	40	I have directly rewarded or supported music creators on short video platforms.	0.789
	41	Short videos make me more willing to pay for digital albums or singles.	0.715
	42	I will follow the social media accounts of recommended musicians in short videos, such as Weibo and Instagram.	0.757
	43	Short videos have shortened my time from ‘hearing music'to‘ deciding to consume'.	0.823
Social identity	44	I feel a sense of belonging when using the same BGM as my friends on SVPs	0.798
	45	My musical preferences on SVPs make me feel recognized by my peers	0.846
	46	Participating in music challenges on SVPs strengthens my connection with other users	0.831
	47	I regard my musical taste on SVPs as an important part of my personal identity	0.809
	48	I am more willing to consume music that is popular among my social circle on SVPs	0.765

All standardized factor loadings range from 0.682 to 0.854, meeting the threshold of ≥0.6 for acceptable construct validity. Data are derived from confirmatory factor analysis (CFA) of valid samples (*n* = 602).

### 3.2 Formal research

The questionnaire was distributed through a combination of online and offline methods to ensure sample representativeness:

(1) Online distribution: Questionnaires were distributed to college students via social media platforms (WeChat, QQ, Weibo), music-related forums, and the professional third-party platform Questionnaire Star. We used snowball sampling to expand coverage across different regions and universities.
(2) Offline distribution: Questionnaires were distributed to undergraduate students from 3 universities in Sichuan provinces. The author (Yu Shan) is a music teacher at Xihua University, with part-time teaching positions at Sichuan University of Culture and Arts and Geely University, where she delivers the course ‘Music Appreciation'.This provided her with convenience in conducting the survey questionnaire. Only students who reported using SVPs to listen to music at least 3 times a week were invited to participate, to avoid non-target responses.

Among the 602 valid questionnaires, 421 were collected online (70.0%) and 181 offline (30.0%). Chi-square tests comparing demographic characteristics (gender, age, grade, monthly household income) between online and offline respondents revealed no significant differences (all p > 0.05), confirming sample representativeness (as shown in Appendix Table 2). Monthly household income was converted to US dollars based on the exchange rate (1 USD ≈ 7 CNY) at the time of data collection: ≤ 6,000 yuan ≈ ≤ 857 USD; 6,001-10,000 yuan ≈ 858-1,429 USD; 10,001-15,000 yuan ≈ 1,430-2,143 USD; ≥15,001 yuan ≈ ≥2,144 USD. The combination of online and offline distribution aimed to expand coverage across different universities and regions in Sichuan Province, rather than altering the target population (college students). Chi-square tests confirmed no significant differences in demographic characteristics between the two samples (all *p* > 0.05), supporting sample representativeness.

The questionnaire was distributed from March 30 to April 11, 2025, with a total of 650 questionnaires collected. Invalid questionnaires were excluded based on the following criteria: (1) response time < 120 s; (2) incomplete mandatory information; (3) identical answers to all Likert-scale questions (e.g., all “4” or “5”). Finally, 602 valid questionnaires were obtained, with an effective response rate of 92.6%. The study protocol was reviewed and approved by the Institutional Ethics Committee of Xihua University. Informed consent was obtained from all participating subjects prior to their involvement in the study. Valid data were analyzed using SPSS 23.0 and AMOS 21.0. First, reverse scoring was performed on the negative item of “algorithm perception” (Q32: “I feel that the content recommended by algorithms is becoming increasingly homogeneous”), and then the following statistical methods were used:

(1) Descriptive statistical analysis: Describe the demographic characteristics of the sample and the distribution of key variables (mean, standard deviation).
(2) Reliability and validity testing: Cronbach's α coefficient for reliability; KMO test, Bartlett's test of sphericity, exploratory factor analysis (EFA), and confirmatory factor analysis (CFA) for validity (convergent validity was evaluated by composite reliability [CR] > 0.7 and average variance extracted [AVE] > 0.5; discriminant validity was evaluated by comparing the square root of AVE with inter-variable correlations). A pilot test was conducted with 80 college students prior to formal data collection. No items were eliminated as all factor loadings exceeded 0.6 (the threshold for item retention). The standardized factor loadings of all items in the formal CFA ranged from 0.682 to 0.897 (see Table 1).
(3) Correlation analysis: Pearson correlation coefficient to test the correlation between variables.
(4) Regression analysis: Multiple linear regression to test the direct effects of independent variables on mediating variables and dependent variables.
(5) Mediation analysis: SPSS PROCESS macro (Model 4 for simple mediation, Model 6 for parallel mediation) with 2000 bootstrap samples to test the mediating roles of music recommendation satisfaction and social identity.
(6) Structural equation modeling (SEM) (Hair et al., 2019): AMOS 21.0 to test the overall fit of the extended SOR model.

This method generates bias-corrected confidence intervals for the indirect effects; an effect is considered statistically significant if the 95% confidence interval does not include zero.

Common method bias test: To mitigate common method variance (CMB), the following procedural measures were adopted: (1) Anonymity was emphasized in the questionnaire to reduce social desirability bias; (2) Item order was randomized to avoid sequence effects; (3) Items measuring different constructs were separated by transition sentences to reduce cognitive associations between variables; (4) Harman's single-factor test was conducted, and results showed that the variance explained by the first common factor was 32.7% (less than 40%), indicating no serious CMB issue in this study.

## 4 Data statistical analysis and result discussion

### 4.1 Descriptive statistical analysis

A frequency analysis of the surveyed university students was conducted based on gender, age, grade, monthly household income, and total daily time spent on short videos. The specific results are shown in Table 2. Basic Information and Frequency Statistics of College Student Users on SVPs.

**Table 2:** Basic information and frequency statistics of college student users on short video platforms.

**Name**	**Option**	**Frequent and continuous**	**Percentage(%)**
Gender	Male	271	45.02
	Female	331	54.98
Age	18-22 years old	512	85.05
	23-25 years old	90	14.95
Grade	Freshman	271	45.02
	Sophomore	120	19.93
	Junior year	151	25.08
	Senior Four	60	9.97
Monthly household income	6,000 yuan and below	295	49
	6,000-10,000 yuan	181	30.07
	10,000-15,000 yuan	76	12.62
	15,000 yuan and above	50	8.31
Total duration of daily use of short videos	Less than 30 min	48	7.97
	30 min to 1 h	133	22.09
	1-3 h	271	45.02
	3-5 h	115	19.1
	More than 5 hours	35	5.81
Total		602	100

It is important to note that this study uses cross-sectional data, and correlations and regression results reflect associations rather than causal relationships. As shown in Table 2, from the perspective of gender, female students accounted for 54.98% of the samples, slightly higher than male students (45.02%), indicating that female university students are the main group of SVP music consumers in the surveyed population. From the perspective of age, 85.05% of the samples were aged 18-22, which is the core age group of undergraduate students and dominates the user base of SVPs. In terms of grade, freshmen accounted for 45.02%, juniors for 25.08%, sophomores for 19.93%, and seniors for 9.97%, with freshmen and juniors forming the main surveyed groups. In terms of monthly household income, the “6,000 yuan or less” category was the most common, accounting for 49%, followed by “6,000-10,000 yuan” (30.07%), reflecting the general economic background of the surveyed college students. In terms of daily SVP usage time, the 1-3 h category accounted for 45.02%, the highest proportion among all time segments, indicating that most university students spend a moderate amount of time on SVPs to access music.

The proportion of college students aged 18-22 in the sample is 85.05%, and the proportion of females is 54.98%. The relatively concentrated age and gender distribution may limit the applicability of research conclusions to other age groups (e.g., graduate students) and male college students. This structural imbalance will reduce the broad representativeness of research results, especially for the explanatory power of short video usage behavior among students over 23 years old and male groups. However, considering that the age range of 18-22 is the main age group for undergraduate students, and that there are already more female users on short video platforms, this sample can still reflect the usage characteristics of core college student users well, so its impact on the overall conclusion is relatively limited.

In the descriptive statistical analysis, in addition to basic information and frequency statistics, the mean and standard deviation were used to measure the level of each variable. A higher mean indicates that the sample has a higher average level for that variable, while the standard deviation describes the dispersion of data in the distribution, such as the size of differences among samples on the same variable. The descriptive statistical results of each research variable are shown in Table 3.

**Table 3:** Descriptive statistical results of each research variable.

**Name**	**Minimum value**	**Maximum value**	**Average value**	**Standard deviation**
Platform usability	1	5	3.51	0.838
Social influence	1	5	3.353	0.789
Emotional regulation	1	5	3.495	0.787
Algorithm Perception	1	5	3.596	0.884
Recommended music satisfaction	1	5	3.532	0.815
Music consumption behavior	1	5	3.122	0.944

Analysis of Table 3 reveals that among the research variables, the average value for algorithm perception is the highest, indicating that university students have a generally positive overall perception of the platform's algorithms, but with significant individual differences; platform usability, emotional regulation, recommended music satisfaction are similar and at an above-average level. There are some differences in university students‘ evaluations of platform usability, while the differences in emotional regulation and recommended music satisfaction are relatively smaller. The average value of social influence is slightly lower, indicating that it plays a relatively weaker role in university students' platform experience, and their perceptions are relatively concentrated. The average value of music consumption behavior is the lowest, indicating that university students do not perform exceptionally well in this aspect and there are significant individual differences.

### 4.2 Reliability and validity testing

#### 4.2.1 Reliability testing

This study used Cronbach's α coefficient to conduct reliability analysis on each measurement scale in the questionnaire, and the results are shown in Table 4.

**Table 4:** Reliability analysis results.

**Dimension**	**Number of items**	**CR**	**AVE**	**Square root of AVE**	**Cronbach's α coefficient**
Platform usability	8	0.935	0.621	0.788	0.922
Social influence	10	0.928	0.593	0.77	0.913
Emotional regulation	10	0.932	0.605	0.778	0.926
Algorithm Perception	4	0.897	0.582	0.763	0.889
Recommended music satisfaction	6	0.901	0.615	0.784	0.922
Music consumption behavior	5	0.93	0.628	0.792	0.872
Social identity	5	0.918	0.603	0.777	0.905

From Table 4, it can be seen that the reliability coefficient values of each dimension are all greater than 0.8, indicating that the reliability quality of the research data is good. Among them, the Cronbach's α coefficients for the dimensions of platform usability, emotional regulation, and satisfaction with recommended music are greater than 0.9, indicating very good reliability quality; the coefficients for social influence, algorithm perception, and music consumption behavior dimensions are also between 0.8 and 0.9, indicating good reliability quality. This reveals that the internal consistency of the measurement scales for each dimension in the questionnaire is high, and the measurement results are stable and reliable, accurately reflecting the true situation of university students in the corresponding dimensions.The square root of AVE for each dimension is greater than the correlation coefficient between the dimension and other dimensions (see Appendix B), indicating good discriminant validity.

#### 4.2.2 Validity testing

The validity testing results based on KMO and Bartlett are shown in Table 5.

**Table 5:** Validity test results based on KMO and Bartlett.

**KMO value**	**Bartlett sphericity test**		
	**Approximate chi-square**	***df***	***p-value***
0.923	11454.64	903	0

From Table 5, it can be seen that the KMO value is 0.923, which is greater than 0.9, indicating that the research data is very suitable for information extraction. At the same time, the Bartlett's test of sphericity *p* value is 0, far less than 0.05, suggesting that there is a significant correlation between the original variables, and all items are suitable for factor analysis. This means that the items in the questionnaire can effectively measure the corresponding variables, the questionnaire has good structural validity, and can accurately reflect the relevant dimensions and characteristics of the impact of SVPs on university students' digital music consumption behavior.

### 4.3 Correlation analysis

Using the Pearson correlation coefficient analysis method, the correlation between variables is tested, where the Pearson correlation coefficient is generally represented by *r*. When *r* is a positive number, it indicates a positive correlation between the variables; when *r* is a negative number, it indicates a negative correlation between the variables. When | *r* | is greater than 0.7, the correlation between the two is very close; when | *r* | is between 0.4-0.7, the two variables are closely related; when | *r* | is between 0.2-0.4, the two variables are weakly related, but there is still a correlation. At the same time, when the significance level is less than 0.05, it indicates that there is a correlation between the two variables. Therefore, the Pearson correlation coefficient is used to study the correlation between music consumption behavior and platform usability, social influence, emotional regulation, algorithm perception, and music recommendation satisfaction, representing the strength of the correlation between variables, as shown in Table 6.

**Table 6:** Pearson correlation analysis results between variables.

**Pearson correlation coefficient**	**Music consumption behavior**	**Platform usability**	**Social influence**	**Emotional regulation**	**Algorithm Perception**	**Recommended music satisfaction**
Music consumption behavior	1					
Platform usability	0.443^***^	1				
Social influence	0.524^***^	0.357^***^	1			
Emotional regulation	0.482^***^	0.451^***^	0.439^***^	1		
Algorithm Perception	0.485^***^	0.445^***^	0.365^***^	0.453^***^	1	
Recommended music satisfaction	0.499^***^	0.418^***^	0.420^***^	0.486^***^	0.378^***^	1

*P* < 0.05; ^**^p < 0.01; ^***^p < 0.001.

From Table 6, it can be seen that music consumption behavior shows significant correlations with platform usability, social influence, emotional regulation, algorithm perception, and satisfaction with recommended music, with correlation coefficients of 0.443, 0.524, 0.482, 0.485, and 0.499 respectively. All correlation coefficients range between 0.443 and 0.524, indicating a moderate-to-strong positive correlation (Cohen, 1988). This suggests that platform usability, social influence, emotional regulation, algorithm perception, and music recommendation satisfaction interact collaboratively to influence college students' digital music consumption behavior.

### 4.4 Regression analysis

Using platform usability, social influence, emotional regulation, and algorithm perception as independent variables, and satisfaction with recommended music as a mediating variable, a linear regression analysis was conducted, with results shown in Table 7.

**Table 7:** Linear regression analysis results of independent variables on mediating variables.

**Variable**	**Non standardized coefficient**		**Standardized coefficient**	***t***	***p***	**Collinearity diagnosis**	
	***B***	**Standard error**	***Beta***			**VIF**	**Tolerance**
Constant	0.910	0.197	-	4.616	0.000^***^	-	-
Platform usability	0.174	0.049	0.179	3.579	0.000^***^	1.412	0.708
Social influence	0.206	0.05	0.199	4.114	0.000^***^	1.322	0.756
Emotional Regulation	0.280	0.054	0.271	5.228	0.000^***^	1.512	0.662
Algorithm Perception	0.095	0.046	0.103	2.047	0.041^*^	1.421	0.704
*R* ^2^	0.329						
Adjust *R*^2^	0.322						
*F*	*F*_(4, 597) =_ 46.332, *p* = 0.000						

Dependent variable = satisfaction with recommended music; ^*^p < 0.05, ^**^p < 0.01, ^***^p < 0.001.

As shown in Table 7, the satisfaction with recommended music is calculated as 0.910 + 0.174 × platform usability + 0.206 × social influence + 0.280 × emotional regulation + 0.095 × algorithm perception. The model's *R*^2^ value is 0.329, indicating that platform usability, social influence, emotional regulation, and algorithm perception can explain 32.9% of the variation in satisfaction with recommended music. When performing an F-test on the model, it was found that the model passed the F-test (F = 46.332, p = 0.000 < 0.05), indicating that at least one of platform usability, social influence, emotional regulation, and algorithm perception has an impact on recommended music satisfaction. Additionally, when testing for multicollinearity in the model, it was found that all VIF values were less than 5, meaning there is no multicollinearity issue, the model is well-fitted. Further analysis reveals: The regression coefficient β for platform usability is 0.174 (*t* = 3.579, *p* = 0.000 < 0.01), indicating that platform usability has a significant positive impact on music recommendation satisfaction. The regression coefficient β for social influence is 0.206 (*t* = 4.114, p = 0.000 < 0.01), indicating that social influence has a significant positive impact on satisfaction with recommended music. The regression coefficient β for emotional regulation is 0.280 (*t* = 5.228, *p* = 0.000 < 0.01), indicating that emotional regulation is significantly positively associated with. music recommendation satisfaction. The regression coefficient β for algorithm perception is 0.095 (*t* = 2.047, *p* = 0.041 < 0.05), indicating that algorithm perception has a significant positive influence on recommended music satisfaction.

Using platform usability, social influence, emotional regulation, algorithm perception, and recommended music satisfaction as independent variables, and music consumption behavior as the dependent variable, a linear regression analysis was conducted, with the results shown in Table 8.

**Table 8:** Results of linear regression analysis of independent variables and mediators on dependent variables (n = 602).

**Variable**	**Non standardized coefficient**		**Standardized coefficient**	***t***	***p***	**95% Confidence interval**	**Collinearity diagnosis**
	***B***	**Standard error**	***Beta***				**VIF**
Constant	−0.612	0.235	-	−2.604	0.009^*^	−1.074,−0.150	-
Platform usability	0.128	0.055	0.112	2.327	0.020^*^	0.035, 0.189	1.482
Social influence	0.215	0.058	0.182	3.707	0.000^***^	0.105, 0.259	1.395
Emotional regulation	0.139	0.061	0.115	2.279	0.023^*^	0.038, 0.192	1.634
Algorithm Perception	0.203	0.052	0.189	3.894	0.000^***^	0.112, 0.266	1.451
Recommended Music satisfaction	0.221	0.057	0.192	3.877	0.000^***^	0.115, 0.269	1.503
*R* ^2^	0.582						
Adjust *R*^2^	0.575						
*F*	*F*_(6, 595)_ = 85.326, *p* = 0.000						

Dependent variable = digital music consumption behavior; ^*^p < 0.05; ^**^p < 0.01; ^***^p < 0.001.

Comparing the effect sizes of the four stimulus variables on digital music consumption behavior, social influence [β = 0.182, 95% CI (0.105, 0.259)] and algorithm perception [β = 0.189, 95% CI (0.112, 0.266)] exhibit relatively larger effect sizes, followed by emotional regulation [β = 0.115, 95% CI (0.038, 0.192)] and platform usability [β = 0.112, 95% CI (0.035, 0.189)]. This indicates that social factors and algorithmic technology exert a more prominent impact on college students' digital music consumption.

As shown in Table 8, music consumption behavior = −0.612 + 0.128 × platform usability + 0.215 × social influence + 0.139 × emotional regulation + 0.203 × algorithm perception + 0.221 × recommended music satisfaction, with an *R*^2^ value of 0.582 and an adjusted *R*^2^ of 0.575. This indicates that platform usability, social influence, emotional regulation, algorithm perception, and music recommendation satisfaction can explain 58.2% of the variation in music consumption behavior. When performing an F-test on the model, it was found that the model passed the F-test (*F* = 85.326, *p* = 0.000 < 0.05), indicating that at least one of the above variables has a significant influence on music consumption behavior. Additionally, multicollinearity was tested for the model, and all VIF values were less than 5, indicating no multicollinearity issues and a well-fitted model. The final analysis reveals: Platform usability significantly predicts music consumption behavior (β = 0.112, *t* = 2.327, *p* = 0.020 < 0.05). The prominence of social influence (β = 0.182, *t* = 3.707, *p* = 0.000 < 0.001) aligns with the core tenets of social identity theory and normative social influence, suggesting that music consumption among university students serves not only a personal hedonic function but also a critical role in social bonding and identity expression within digital communities. The desire to affiliate with peers and adhere to group norms can transcend individual taste, directly motivating consumption to signal belonging and shared identity, thus suggesting that social influence is related to the predictive factors in the model. Emotional regulation was a significant positive predictor of music consumption behavior (β = 0.115, *t* = 2.279, *p* = 0.023 < 0.05). Algorithm perception has a significant positive influence on music consumption behavior (β = 0.189, *t* = 3.894, *p* = 0.000 < 0.001). Recommended music satisfaction also exerts a significant positive influence on music consumption behavior (β = 0.192, *t* = 3.877, *p* = 0.000 < 0.001).

Although the model explained 58.2% of the variation in music consumption behavior, 41.8% of the variation was not included in the explanation, indicating the presence of other potential influencing factors. Personal music preferences may play an important role, such as a preference for specific music styles or artist loyalty, which directly affects consumer decisions. Economic ability is also a key variable, and college students with different consumption abilities may exhibit differentiated willingness and behavior to pay. In addition, social and cultural factors such as cultural background, education level, and participation in offline music activities may also have an impact. Differences in technological acceptance, such as adaptability to emerging music technology products, may also regulate consumer behavior. These unmeasured variables constitute complex influencing factors outside the model, and future research can further optimize explanatory power by expanding the variable system or introducing moderating effects. It is worth noting that a 45% explanatory rate in the field of social sciences is already at a reasonable level, indicating that the current model has captured the core influencing factors.

### 4.5 Structural model and mediation analysis

**Figure 2:**
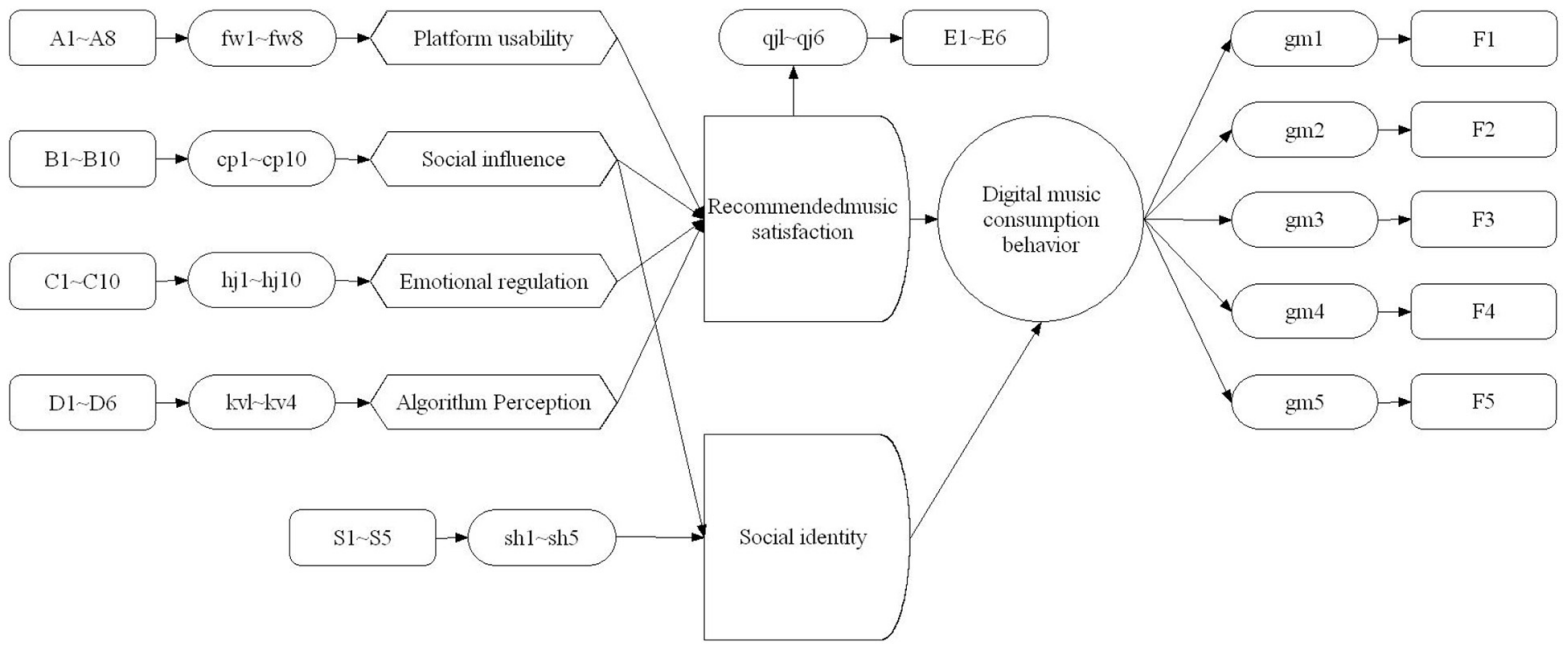
Structural model diagram.

To empirically test the complete SOR model and the hypothesized mediating pathways illustrated in Figure 1, a structural equation modeling (SEM) analysis was conducted. The hypothesized structural model is presented in Figure 2. Analysis using AMOS 21.0 showed that the model demonstrated a good fit to the data: χ^2^/df = 1.415, GFI = 0.952, CFI = 0.988, RMSEA = 0.032, indicating that the theoretical structure is well-supported.

An alternative model was tested where social identity mediates the relationship between platform usability and consumption behavior. Fit indices of the alternative model (χ^2^/df = 2.135, GFI = 0.897, CFI = 0.923, RMSEA = 0.058) are worse than the original model, confirming the original model's superiority. SEM provides additional value beyond regression by simultaneously testing direct and indirect effects, allowing for comprehensive validation of the theoretical framework.

The significance of the mediation effects was tested using the bootstrap method with 2,000 samples. The results confirmed that both music recommendation satisfaction and social identity served as significant mediators:

(1) Music recommendation satisfaction mediated the relationships between platform usability [Standardized indirect effect = 0.202, 95% CI (0.146, 0.259)], emotional regulation [Standardized indirect effect = 0.207, 95% CI (0.159, 0.253)], algorithm perception [Standardized indirect effect = 0.183, 95% CI (0.135, 0.231)] and digital music consumption behavior.
(2) Social identity mediated the relationship between social influence and digital music consumption behavior [Standardized indirect effect = 0.245, 95% CI (0.187, 0.302)].
(3) The indirect effect of social influence through music recommendation satisfaction was not significant [Standardized indirect effect = 0.005, 95% CI (−0.031, 0.033)], confirming that social influence primarily exerts its effect through social identity.

This non-significant mediation pathway (social influence → music recommendation satisfaction → consumption behavior) can be explained by the social nature of music consumption among college students. When influenced by peers, celebrities, or music charts, college students prioritize the “social value” of music (e.g., whether it can help them integrate into peer groups, gain recognition, or participate in collective activities like music challenges) rather than its “individual preference match.” Thus, social influence drives consumption through the formation of social identity (a collective psychological state) rather than individual satisfaction with recommended music. In contrast, platform usability, emotional regulation, and algorithm perception are closely related to individual interaction experiences with the platform, so their impact on consumption is mediated by personal evaluation of recommendation quality.

These findings robustly support the proposed SOR framework, delineating both the direct psychological pathways and the key mediating role of internal satisfaction in translating platform stimuli into consumption behavior. The significant mediating role of recommendation satisfaction underscores the importance of internal, cognitive-affective evaluations in the consumption pathway, aligning with theories of technology acceptance that emphasize perceived usefulness and satisfaction as key drivers of behavior (e.g., the Expectation-Confirmation Model). Furthermore, the dominant direct effect of social influence highlights that in highly networked environments like SVPs, consumption is not merely an individual choice but a socially situated behavior, resonating with principles of Social Identity Theory. This suggests that the SOR model's “Organism” component can be effectively unpacked into both individualistic (satisfaction) and collectivistic (social identity) psychological processes.

### 4.6 Moderating effect analysis

Hierarchical regression analysis was conducted to test the moderating effect of social identity. Results show that the interaction term between music recommendation satisfaction and social identity is significant (β = 0.123, *p* < 0.01), indicating that social identity positively moderates the relationship. For students with high social identity (M + 1SD), the association between recommendation satisfaction and consumption behavior is stronger (β = 0.285, *p* < 0.001) than for those with low social identity (M - 1SD, β = 0.162, *p* < 0.001).

## 5 Discussion and conclusion

Grounded in the Stimulus-Organism-Response (SOR) framework (Bagozzi and Warshaw, 1990), this study delineates the psychological mechanisms through which short video platform (SVP) engagement shapes the digital music consumption behaviors of university students. The empirical results robustly support the proposed model, revealing a complex interplay between platform stimuli, internal cognitive-affective states, and consequential consumption behaviors. The following sections discuss the theoretical contributions, practical implications, and avenues for future research arising from these findings.

### 5.1 Theoretical Implications

This study makes three key theoretical contributions: First, it refines the SOR framework in a context-specific manner by integrating social identity as a parallel mediator alongside music recommendation satisfaction, thereby extending the “Organism” component from a predominantly individual-cognitive focus to a dual structure encompassing both individual cognition and social cognition. This refinement addresses the context-specific limitation of existing SOR studies that overlook the social nature of young consumers' music behavior (Park and Jung, 2024), distinct from Nario-Redmond et al. (2004)'s broad cross-domain social identity measurement by focusing on SVP music consumption-specific psychological mechanisms. Unlike prior studies that treat the “Organism” as a unidimensional construct, our dual-mediator design captures both individual and collective psychological processes, providing a more nuanced understanding of SVP-driven music consumption.

Second, it clarifies the differentiated mediating pathways of different stimulus factors: platform usability, emotional regulation, and algorithm perception correlate with consumption through music recommendation satisfaction (individual pathway), while social influence correlates with consumption through social identity (social pathway). Comparing the effect sizes of the four stimulus variables on digital music consumption behavior, social influence [β = 0.182, 95% CI (0.105, 0.259)] and algorithm perception [β = 0.189, 95% CI (0.112, 0.266)] exhibit relatively larger effect sizes, followed by emotional regulation [β = 0.115, 95% CI (0.038, 0.192)] and platform usability [β = 0.112, 95% CI (0.035, 0.189)]. This indicates that social factors and algorithmic technology exert a more prominent impact on college students' digital music consumption, aligning with the social embedding characteristic of SVP usage. The non-significant path from social influence to music recommendation satisfaction further confirms the uniqueness of the social pathway—when influenced by peers, celebrities, or music charts, undergraduate students prioritize the “social value” of music (e.g., group integration, recognition) over individual preference matching, which resonates with social identity theory (Tajfel and Turner, 1979) and distinguishes our findings from studies focusing solely on individual satisfaction (Ning et al., 2022).

Third, it supplements the literature on digital music consumption by confirming social identity as a powerful driver and revealing its moderating role in the relationship between music recommendation satisfaction and consumption behavior. The moderating effect (β = 0.123, *p* < 0.01) indicates that the interplay between individual satisfaction and social identity enhances the prediction of consumption behavior—students with higher social identity are more likely to translate recommendation satisfaction into consumption, as music not only meets personal preferences but also reinforces group identity. This finding extends Maccarrone-Eaglen and Schofield (2023)'s research on social identity in social media consumption by demonstrating both mediating and moderating effects of social identity in the SVP music context. Additionally, our study validates that the SOR framework's “Organism” component can be effectively unpacked into individualistic (satisfaction) and collectivistic (social identity) psychological processes, addressing the gap identified by Park and Jung (2024) in their review of SOR applications in digital media.

While platform usability, emotional regulation, and algorithm perception operate through both direct and satisfaction-mediated pathways, social influence functions primarily through a direct route to consumption. This critical finding suggests that not all platform stimuli are processed through the same psychological channel. It challenges overly simplistic mediation models and calls for a more differentiated understanding of how different platform features “hook” users, with social drivers potentially bypassing deep cognitive evaluation in favor of normative and impulsive responses.

### 5.2 Practical implications

The findings offer actionable insights for SVP operators and music industry stakeholders.

For SVP operators, the significant mediating role of recommendation satisfaction highlights the need to continuously refine algorithmic accuracy and diversity. Platforms should not merely push popular content but also develop “deep discovery” modules that intelligently recommend professional artists and the creative narratives behind music styles to users who have demonstrated sustained interest. At the same time, platforms must address the risks of information cocoons and creative homogenization that algorithms may cause, integrating ethical considerations into their design. Based on the mediating role of social identity, SVPs should develop identity-centered community features, such as music interest groups, personalized member badges, and collaborative creation tools. These features can enhance users' sense of belonging and social identity, thereby promoting digital music consumption.

For the music industry and artists, while recognizing SVPs as powerful drivers of discovery and commerce, a more critical and strategic approach to engagement is necessary. First, when leveraging social influence for marketing campaigns or tracking BGM trends, caution must be exercised to avoid over-reliance on viral exposure, which could undermine the long-term development of artistic identity and sustainability. Second, artists and labels should proactively establish institutionalized dialogues with SVP platforms to negotiate issues such as fair and transparent revenue-sharing models, transparency in algorithmic logic, and feature designs that support artistic expression. This will help ensure that platform mechanisms better serve artists' diverse goals and identity formation.

For university education and guidance, understanding the profound influence of social factors and algorithmic curation on music consumption can inform the design of effective media literacy programs. Educators should guide students to recognize the role of social identity in consumption behaviors and foster rational consumption attitudes (Humphry et al., 2024), with particular attention to impulsive consumption driven by peer pressure or group conformity. Media literacy initiatives should not only promote rational consumption but also raise awareness of the potential risks of excessive social influence. Furthermore, they should encourage critical reflection on deeper issues, such as how algorithms shape cultural diversity and the working conditions of artists.

### 5.3 Limitations and future research

This study has three limitations that can be addressed in future research: First, the sample is limited to undergraduate students from three universities in Sichuan Province, China, and results may not be generalizable to graduate students, students from other regions/countries, or non-student populations. Future studies should expand the sample to include more regions, age groups, and cultural backgrounds to improve generalizability. Second, the cross-sectional design prevents causal inference, and mediation results should be interpreted as tentative associations rather than causal relationships. The mediating pathways identified in this study provide a theoretical foundation, but longitudinal designs are needed to verify the temporal order of variables and confirm causal mechanisms. Third, all variables were measured via self-report at a single time point, which may inflate relationships due to common method variance. Although Harman's single-factor test showed no serious CMB (variance explained by the first factor = 32.7%), future studies should adopt multi-source or multi-wave data collection to further mitigate this issue. Fourth, while the extended SOR model explains 58.2% of the variance in consumption behavior, other potential factors (e.g., individual personality traits, economic capacity, music genre preferences) are not included. Future studies could add these variables as moderators or control variables to further improve the model's explanatory power. Fifth, one measurement-related limitation concerns the potential for double-barreled item phrasing in the survey. For instance, the item assessing platform usability stated, “The platform's music search function (such as humming recognition) is accurate and efficient.” While this phrasing combines two conceptually related aspects of feature performance—where “efficiency” without “accuracy” would be of limited utility in a search context—it risks conflating distinct evaluations. To empirically assess whether this design choice meaningfully biased our results, we conducted a *post-hoc* sensitivity analysis. The item in question was temporarily removed from the “Platform Usability” scale, and all key analyses (reliability, validity, structural model testing) were re-run. Results indicated that the scale's reliability remained high (Cronbach's α > 0.91), construct validity was preserved, and—most critically—the standardized path coefficients from platform usability to both music recommendation satisfaction and digital music consumption behavior remained statistically significant and substantively unchanged in magnitude. This suggests that while the phrasing could be optimized in future studies (e.g., by separating “accuracy” and “efficiency” into distinct items), its presence did not distort the fundamental relationships reported in this research. Future work should employ more granular item formulations to further enhance measurement precision.

Future research could incorporate additional variables such as individual personality traits (e.g., openness to experience, impulsivity), specific music genres, economic capacity, and measures of actual listening behavior (e.g., streaming time) to build a more comprehensive model.

In conclusion, this study successfully maps the psychological journey from SVP engagement to music consumption, validating and refining the SOR framework. It highlights the critical roles of both internal satisfaction and external social forces, as well as the moderating effect of social identity, providing a solid foundation for both future academic inquiry and strategic practice in the evolving digital music ecosystem. The non-significant indirect effect of social influence through recommendation satisfaction highlights the uniqueness of the social pathway—students prioritize the social value of music (e.g., group integration) over individual preference matching when influenced by social factors.

## Data Availability

The raw data supporting the conclusions of this article will be made available by the authors, without undue reservation.
