# Supplementary material for: Driven by engagement and social identity: refining the SOR framework to explore digital music consumption behaviors among Chinese undergraduate students on short video platforms
Source: Front Psychol. 2026 Mar 6;17:1758762. doi: 10.3389/fpsyg.2026.1758762 (PMC13002847; doi:10.3389/fpsyg.2026.1758762)
Supplement: Supplementary file 1 [file Supplementary_file_1.docx]

**Appendix A**

**TABLE A1. Results of bootstrap mediation analysis for the structural model**

| **Mediating effect pathway** | **Standardized mediation effect value** | **Total indirect effect** | **Bootstrap lower limit (95% CI)** | **Bootstrap upper limit (95% CI)** | **Significance** |
| --- | --- | --- | --- | --- | --- |
| Platform usability - Recommended music satisfaction - Digital music consumption behavior | 0.202 | 0.330 | 0.146 | 0.259 | Significant (CI does not include 0) |
| Social influence - Recommended music satisfaction - Digital music consumption behavior | 0.005 | 0.427 | -0.031 | 0.033 | Not significant (CI includes 0) |
| Emotional regulation - Recommended music satisfaction - Digital music consumption behavior | 0.207 | 0.346 | 0.159 | 0.253 | Significant (CI does not include 0) |
| Algorithm Perception - Recommended Music Satisfaction - Digital Music Consumption Behavior | 0.183 | 0.392 | 0.135 | 0.231 | Significant (CI does not include 0) |

*Note: The overall model fit indices are χ²/df=1.415, GFI=0.952, CFI=0.988, RMSEA=0.032, indicating a good model fit.*

**TABLE A2. Comparison of demographic characteristics between online and offline respondents**

| **Name** | **Option** | **Online Respondents (n=421)** | **Offline Respondents (n=181)** |
| --- | --- | --- | --- |
| Gender | Male | 189 (44.9%) | 82 (45.3%) |
|  | Female | 232 (55.1%) | 99 (54.7%) |
| Age | 18-22 years old | 359 (85.3%) | 153 (84.5%) |
|  | 23-25 years old | 62 (14.7%) | 28 (15.5%) |
| Grade | Freshman | 189 (45.1%) | 81 (44.8%) |
|  | Sophomore | 84 (20.0%) | 36 (19.9%) |
|  | Junior year | 105 (24.9%) | 46 (25.4%) |
|  | Senior Four | 43 (10.2%) | 18 (9.9%) |
| Monthly household income | 6000 yuan and below | 206 (48.9%) | 89 (49.2%) |
|  | 6000-10000 yuan | 127 (30.2%) | 54 (29.8%) |
|  | 10000-15000 yuan | 53 (12.6%) | 23 (12.7%) |
|  | 15000 yuan and above | 35 (8.3%) | 15 (8.3%) |
| Total duration of daily use of short videos | Less than 30 minutes | 15 (8.3%) | 15 (8.3%) |
|  | 30 minutes to 1 hour | 93 (22.1%) | 40 (22.1%) |
|  | 1-3 hours | 189 (44.9%) | 82 (45.3%) |
|  | 3-5 hours | 81 (19.2%) | 34 (18.8%) |
|  | More than 5 hours | 25 (5.9%) | 10 (5.5%) |

*Note: 1. χ² test was used to compare differences between online and offline respondents; 2. p > 0.05 indicates no significant demographic differences between the two groups, confirming sample representativeness; 3. Monthly household income converted to US dollars (1 USD ≈ 7 CNY) for reference: ≤6,000 CNY ≈ ≤857 USD, 6,001–10,000 CNY ≈ 858–1,429 USD, 10,001–15,000 CNY ≈ 1,430–2,143 USD, ≥15,001 CNY ≈ ≥2,144 USD.*

**Appendix B**

**TABLE B1. Correlation matrix and discriminant validity test results**

| **Dimension** | **1** | **2** | **3** | **4** | **5** | **6** | **7** |
| --- | --- | --- | --- | --- | --- | --- | --- |
| 1. Platform usability | 0.788¹ |  |  |  |  |  |  |
| 2. Social influence | 0.357*** | 0.770¹ |  |  |  |  |  |
| 3. Emotional regulation | 0.451*** | 0.439*** | 0.778¹ |  |  |  |  |
| 4. Algorithm Perception | 0.445*** | 0.365*** | 0.453*** | 0.763¹ |  |  |  |
| 5. Recommended music satisfaction | 0.418*** | 0.420*** | 0.486*** | 0.378*** | 0.792¹ |  |  |
| 6. Digital music consumption behavior | 0.443*** | 0.524*** | 0.482*** | 0.485*** | 0.499*** | 0.759¹ |  |
| 7. Social identity² | 0.289*** | 0.632*** | 0.345*** | 0.298*** | 0.312*** | 0.476*** | 0.784¹ |
